# The Intersection of COVID-19 and Rheumatoid Arthritis: Shared Mechanisms, Treatment Challenges, and Potential Therapeutic Approaches

**DOI:** 10.14336/AD.2023.0406

**Published:** 2023-08-01

**Authors:** Jie Xiao, Chaoyi Liang, Lizeai Zhang, Jinling Li, Sijia Liu

**Affiliations:** ^1^Guangxi Colleges and Universities Key Laboratory of Biological Molecular Medicine Research, Department of Biochemistry and Molecular Biology, School of Basic Medical Sciences, Guangxi Medical University, Nanning, Guangxi, China.; ^2^Collaborative Innovation Centre of Regenerative Medicine and Medical BioResource Development and Application Co-constructed by the Province and Ministry, Guangxi Key Laboratory of Regenerative Medicine & Key Laboratory of Longevity and Aging-related Diseases of Chinese Ministry of Education, Guangxi Medical University, Nanning, Guangxi, China.; ^3^Laboratory of Basic Medicine Center, Guangxi Medical University, Nanning, Guangxi, China

**Keywords:** COVID-19, rheumatoid arthritis, cytokine storm, therapeutics

## Abstract

Severe acute respiratory syndrome coronavirus 2 (SARS-CoV-2) is spreading rapidly across the world, posing a major health concern with the coronavirus disease 2019 (COVID-19). Patients with rheumatoid arthritis (RA) may be at higher risk of infection and disease progression due to impaired autoimmune systems, immunosuppressants, and comorbidities. Therefore, we review the possible immune mechanisms and pathological interactions between COVID-19 and RA, as the uncontrolled immune activation and cytokine response in COVID-19 resemble the immune inflammation of RA. We also discuss the potential mechanisms that may lead to cardiovascular complications as well as the challenges of treating RA patients with COVID-19. While several therapeutic agents are being developed to cure COVID-19, antirheumatic drugs could also be potential options due to the similar proinflammatory cytokines induced in both diseases. Additionally, we discuss the safety and effectiveness of SARS-CoV-2 vaccines and novel therapeutic approaches against RA based on the shared mechanisms between COVID-19 and RA.

## 1. Introduction

Infections caused by SARS-CoV-2 are primarily transmitted through respiratory droplets and close contact, which are highly pathogenic and transmissible [1]. COVID-19 caused by SARS-CoV-2 is a severe respiratory disease that affects multiple populations, especially the elderly, and spreads rapidly worldwide, posing a serious threat to human beings. According to World Health Organization (WHO) statistics (https://covid19.who.int/), there have been over 760 million confirmed cases worldwide so far, with nearly 6.8 million deaths. However, there is still no definitive explanation for the pathogenesis of COVID-19. It is currently believed that cytokine regulation disorders and high inflammation are responsible for the main clinical manifestations and complications of this disease, which are called cytokine storms. After SARS-CoV-2 infection, a large number of signal responses are induced within the lungs, followed by the release of cytokines and chemokines such as IL-1β, IL-6, IL-8, CXCL10, and TNF-α, and the recruitment of inflammatory cells to the infected sites, which often leads to acute respiratory distress syndrome (ARDS), multiple organ failure, and even death [2, 3]. It is noteworthy that the cytokine and immune activation patterns in COVID-19 patients appear similar to those in patients with RA, a chronic systemic autoimmune disease that affects multiple joints and is often accompanied by synovitis characterized by cell infiltration, synovial tissue proliferation, angiogenesis, and cartilage damage [4, 5]. In the local inflammatory sites of RA patients, immune cells and proinflammatory cytokines, including IL-1β, IL-6, IL-17, and TNF-α, play a crucial role in the pathogenesis by regulating inflammation, autoimmunity, and joint destruction [6]. Therefore, there seems to be a possibility of shared pathological cascades occurring in both COVID-19 and RA.

In RA patients, the anti-infection ability may be lower than that of the general population due to potential immune system dysfunction, comorbidities, and long-term immunosuppressive medication [7, 8]. According to some previous studies, COVID-19 occurs frequently in patients with RA, and the symptoms are more severe than those in the general population [9]. Moreover, the immune changes directly caused by RA or the indirect effects of treatments may lead to a poor prognosis for COVID-19 [10]. As a result, the high possibility of infection with SARS-CoV-2 and the adverse outcome of COVID-19 undeniably pose a significant obstacle to the treatment of RA patients [11].

Converging studies on COVID-19 indicate that some drugs used for the treatment of RA can also act on COVID-19 [12]. However, it is still unknown which mechanisms underlie the relationship between RA and COVID-19. Therefore, we introduce the epidemiology of COVID-19 and RA, elaborate on the similar pathogenesis underlying them, as well as the challenges associated with treating RA patients during the COVID-19 outbreak, and shed light on potential treatment strategies.

## 2. Epidemiology and Clinical Characteristics of COVID-19 in RA Patients

### 2.1 Epidemiology of COVID-19 in RA patients

Previous studies have shown that infectious diseases are the leading cause of premature death in RA patients [13-16]. RA patients have a 3.5 times higher risk of infection than the general population, according to a prospective cohort study conducted in the UK [17]. The hyperactivity of RA disease is associated with an increased risk of infection [18, 19]. RA patients have increased thymus dysfunction, peripheral T cell renewal, and circulatory T cell dysfunction, making them more vulnerable to viral infections [20].

Since the COVID-19 pandemic began, there have been numerous cases of COVID-19 diagnosed in rheumatic patients. An analysis of seven case-control studies reported that the incidence rate of COVID-19 in patients with autoimmune diseases was twice that in the general population [21]. In a recent large-scale study, researchers compared patients with and without RA and found that RA sufferers had a 25% increased chance of contracting SARS-CoV-2 and a 35% increased risk of dying or requiring medical attention as a result of COVID-19 [9]. An early comparative cohort study conducted in Boston, Massachusetts, also suggested that rheumatic disease patients with COVID-19 were more likely to require mechanical ventilation than those in the control group [22]. In a comparative cohort study conducted by D'Silva et al., which included 52 rheumatic patients and 104 non-rheumatic patients with COVID-19 infection, it was found that patients with and without rheumatism had similar rates of hospitalization and mortality. However, the probability of patients with rheumatism requiring severe care and mechanical ventilation was higher than that of the comparison [23, 24].

### 2.2 Patient characteristics associated with COVID-19 in RA

Most hospitalized patients are older among RA patients. They are more prone to complications such as asthma, chronic obstructive pulmonary diseases, hypertension, diabetes, etc., which further increase the risk of viral infection [25-27]. The OpenSAFELY analysis platform conducted a large-scale analysis of data collected by over 17 million UK citizens, showing that the risk of fatal COVID-19 is greatly increased with the increase in obesity, diabetes, severe asthma, respiratory diseases, chronic heart disease, liver disease, renal dysfunction, and/or autoimmune diseases, etc. [28]. Among 1527 COVID-19 patients, 17.1% had hypertension and 16.4% had heart disease, according to a meta-analysis. They required intensive care more often [29]. COVID-19 mortality and severity were significantly increased by hypertension, according to a retrospective analysis [30-32]. It is noted that an increased likelihood of insulin resistance and type 2 diabetes is seen in patients with RA [33]. There were also signs that patients with diabetes have an increased risk of contracting COVID-19 early in its development [29]. An observational study of 193 patients with severe COVID-19 showed that there was a higher mortality rate in patients with diabetes than in patients without diabetes [22]. At the same time, patients with COVID-19 who suffer from diabetes comorbidity are 14.2% more likely to require intensive care [34], implying a correlation with the high expression of angiotensin-converting enzyme 2 (ACE2) in diabetes patients. In addition, patients with RA are more likely to suffer from venous thromboembolism (VTE) than the general population [35]. As reported by the RA-COVID-19 study, VTE and sepsis were more prevalent among RA patients. Therefore, it is critical to closely monitor the disease dynamics of RA patients with other comorbidities during the prevalence of COVID-19. Observational studies and clinical trials show that some commonly used immunosuppressants for RA may affect the severity of COVID-19 [36], as shown below.

### 2.3 Impact of immunosuppressants on COVID-19 in RA patients

There has been concern that immunosuppressive therapies used to treat RA could increase the risk of severe COVID-19 in patients. However, the evidence is still limited and conflicting [36-38]. A retrospective study of 4,464 COVID-19 patients found no significant difference in disease severity or outcome between patients with RA on biologic disease-modifying antirheumatic drugs (bDMARDs) and those who were not [39]. Similarly, another study found no significant difference in the severity of COVID-19 between patients on conventional synthetic disease-modifying antirheumatic drugs (csDMARDs) and those who were not [40]. However, a study from a French cohort of patients with rheumatic diseases and COVID-19 showed that patients with rheumatic diseases on glucocorticoids, but not other DMARDs, were at higher risk of severe COVID-19 [41].

In contrast, some studies suggest that immuno-suppressive therapies may have a protective effect against severe COVID-19 in RA patients. A physician-reported case registry of 600 RA patients diagnosed with COVID-19 found that patients on bDMARDs had a lower risk of hospitalization due to COVID-19 compared to those who were not [40]. Another study found that RA patients on bDMARDs had a lower risk of severe COVID-19 compared to those who were not on these drugs [42].

Overall, the evidence on the impact of immuno-suppressants on COVID-19 in RA patients is still limited and conflicting. More studies are needed to clarify the association between these drugs and the severity of COVID-19 in RA patients. In the meantime, it is important for RA patients to continue taking their medications as prescribed and to follow public health guidelines to reduce the risk of COVID-19 infection. Close monitoring of RA patients on immunosuppressive therapies is also necessary to ensure early detection and appropriate management of COVID-19.

## 3. Shared Mechanisms of COVID-19 and RA

### 3.1 Similarities between immune response and cytokine dysregulation

COVID-19 activates both innate and adaptive immunity. Innate immunity quickly activates immune cells such as macrophages, natural killer (NK) cells, and others, which produce inflammatory cytokines and chemokines to defend against the viral invasion [43]. Adaptive immunity dispatches CD8+ T cells to identify the structural protein of SARS-CoV-2, with the help of CD4+ T cells and infected alveolar epithelial cells presenting it. Then, CD8+ T cells release pro-apoptotic factors such as granzyme and perforin to promote target cells to programmed cell death. Memory B lymphocyte cells differentiate into plasma cells and produce antibodies under the stimulation of activated Th1 cells [44]. Blocking the attachment between the receptor of susceptible cells and the virus requires binding the antigenic protein of the virus with anti-SARS-CoV-2 antibodies. Generally, the body relies on its immune response to clear the virus, which results in mild and common types of the disease. However, the virus may reach the lungs and activate immune cells to produce inflammatory factors, inducing more immune cells to produce more cytokines at the inflammatory sites. If the protective immune response is damaged, this can form a "positive feedback" cycle. Thus, the uncontrolled surge of pro-inflammatory mediators in the immune system is the reason for the formation of a cytokine storm, causing the development of ARDS. This is considered one of the main reasons why disease development is so rapid, and mortality is so high in patients with COVID-19 [45]. Clinical evidence suggests that patients with severe COVID-19 have markedly increased levels of pro-inflammatory cytokines within their bodies, including IL-1β, IL-2, IL-6, IL-7, IL8, TNF-α, CCL2, MIP-1α, and CXCL10 [2, 45, 46]. The antiviral immune response involving IFN-I and IFN-III, belonging to innate immunity, may be diminished or delayed, resulting in excessive inflammatory responses [47]. Zhou et al. found that upon novel coronavirus infection, CD4+ T lymphocytes promptly changed into pathogenic T helper (Th) 1 cells, and they could secrete GM-CSF. Additionally, inflammatory CD14+CD16+ monocytes expressed large quantities of IL-6 as a result of the highly inflammatory cytokine environment [48]. The lungs of patients with severe COVID-19 are abundant in highly inflammatory macrophages, which secrete inflammatory mediators like IL-1β, IL-6, IL-8, CXCL10, TNF-α, and other chemokines, further exacerbating the occurrence of cytokine storms [49-51]. Naive T cells are derived to convert into mature Th17 cells to produce IL-17 by antigen-presenting cells, recruiting neutrophils to the place where inflammation occurs to activate macrophages to alter the inflammatory environments [52-54]. Studies have shown significantly reduced numbers of T cells, especially CD4+ and CD8+ T cells, within COVID-19 patients, particularly those in intensive care unit (ICU), and the function of surviving T cells is impaired in vivo [55]. Reduced T cell count and functional failure cause adaptive immune response disorders, weaken the immune system, and affect the disease's prognosis [56]. Lung autopsies showed that alveolar exudate contains moderate amounts of macrophages and a small number of neutrophils, while the interstitial lung showed visible T lymphocytes [57].

**Figure 1.**
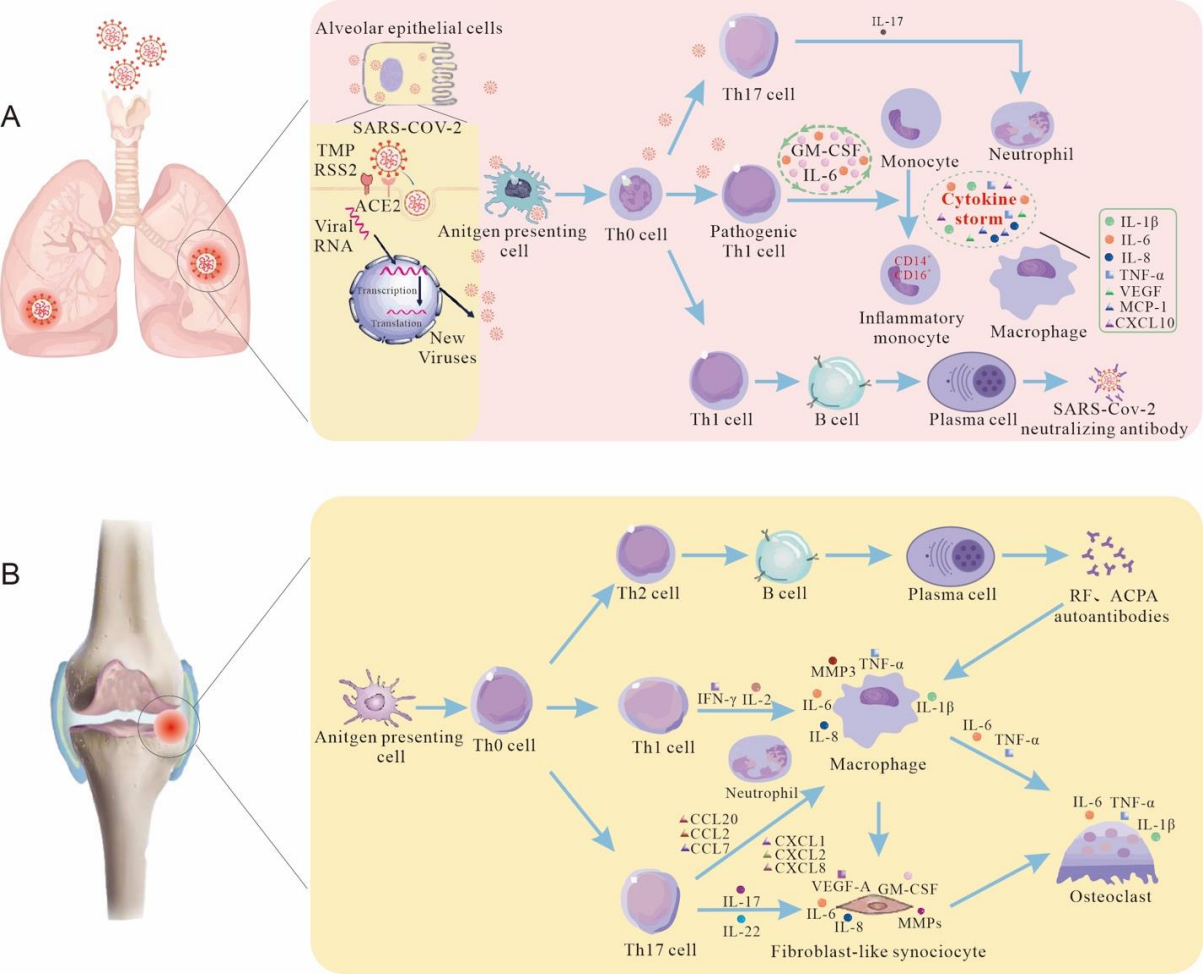
The similar pathophysiological mechanisms between SARS-CoV-2 infection (A) and RA (B).

The development of chronic synovitis in RA is associated with the excessive activation of T cells and an imbalance of inflammatory cytokines. B lymphocytes are activated by continuous antigenic stimulation of antigen presenting cells (APC) and differentiate into plasma cells that secrete immunoglobulins, including rheumatoid factor (RF) and anti-citrullinated peptide antibodies (ACPA). These immunoglobulins form immune complexes with antigens and stimulate macrophages to secrete pro-inflammatory cytokines like TNF-α and IL-6 [58]. Additionally, Th1, Th2, and Th17 cells, which have a close relationship in inflammatory response, are all differentiated from CD4+ T cells and interact with resident macrophages, DC cells, synoviocytes, and osteoclasts to maintain synovial inflammation. Activated Th1 cells can secrete IFN-γ and IL-2, induce differentiation of classically activated macrophages (M1), and further promote cytokine production [59]. Pathogenic Th17 cells are considered positive regulators of the immune response because they produce inflammatory factors like IL-17 and IL-22. IL-17 can induce the production of proinflammatory cytokines from chondrocytes, synovial macrophages, and osteoclasts, such as TNF-α, IL-1β, and IL-6 [60]. It can also stimulate the production of various chemokines, including CXCL1, CXCL2, CXCL8, CCL2, CCL7, and CCL20, which enhance inflammation by recruiting neutrophils, macrophages, and lymphocytes into the synovium [61]. TNF-α, IL-6, and IL-17 can jointly promote further production of cytokines by fibroblast-like synoviocytes (FLS), such as IL-6, IL-8, VEGF-A, GM-CSF, and matrix metalloproteinases (MMP1 and MMP3), leading to chronic synovitis and the destruction of cartilage and bone [58, 62]. Both COVID-19 and RA exhibit an excessive immune response and cytokine imbalance, which are caused by the overactivation of T cells (especially Th1 and Th17 cells) that secrete a large number of cytokines, including TNF-α, IL-1β, IL-6, IL-8, IL-17, and GM-CSF, leading to tissue damage (Fig. 1).

**Figure 2.**
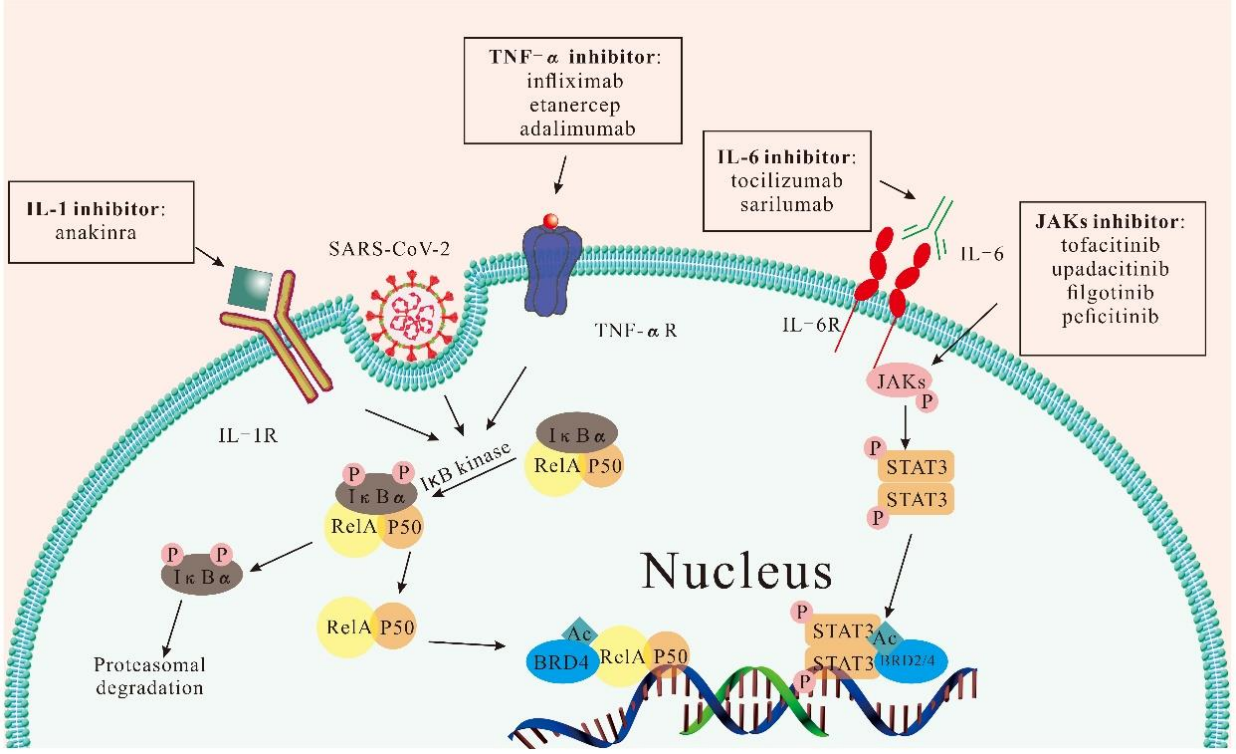
Activation of NF-KB and JAK-STAT signaling pathway by SARS-CoV-2 and the application of RA drugs in anti-SARS-CoV-2 therapy.

As mentioned above, IL-6, IL-1β, and TNF-α are all key cytokines in both COVID-19 and RA. For example, IL-6 activates JAK phosphorylation upon binding to IL-6Rα/gp130 complexes and then recruits and phosphorylates STAT protein to generate p-STAT dimer, which promotes target gene transfer into the nucleus [63]. Activation of the JAK-STAT signaling pathway has been found in both RA and COVID-19 [64, 65]. As it is known, IFN-I is engaged in innate immunity by initiating JAK-STAT signaling to make the organism ready for antiviral pathways [66]. However, the nucleocapsid protein of SARS-CoV-2 probably blocks innate immunity with IFN-I involvement by inhibiting the phosphorylation of STAT1 and STAT2 [67]. NF-κB signals participate in inflammatory responses through complex molecular regulation. IκB kinase is the key to NF-κB pathway activation, and viruses, pro-inflammatory factors, radiation, and Angiotensin II (Ang II) are its activating factors [68]. COVID-19 and RA coexist with TNF-α and IL-1 to activate the NF-κB pathway [62, 69]. Su et al. [70] conducted an in-depth study of the ORF7a protein originating from SARS-CoV-2 and found that it activates the NF-κB pathway and promotes the secretion of pro-inflammatory cytokines such as IL-1α, IL-10, and various chemokines. Another similar study also showed that the spike (S) protein, a structural protein of SARS-CoV-2, induced the activation of NF-κB that can assist in the secretion of inflammatory mediators, including CXCL1, CXCL2, CCL2, IL-6, IL-1β, and TNF-α, among others [71]. Moreover, high levels of Ang II were observed in severe COVID-19 patients, and thus it can be hypothesized that the cytokine storm often seen in this population has the involvement of Ang II-induced NF-κB pathway activation [72]. Therefore, the pathogenesis of COVID-19 and RA is similar in that the inflammatory state in the pathogenesis of both diseases activates the JAK-STAT and NF-κB signaling pathways (Fig. 2), which offers possibilities for the therapy of RA patients infected by SARS-CoV-2.

Neutrophil extracellular trap networks (NETs) are mainly composed of DNA and histones, have a web-like structure, and are released by activated neutrophils [73]. Overproduction of NETs may recruit pro-inflammatory cells and facilitate the generation of autoantibody immune complexes [74, 75]. NETs also appear to be related to COVID-19 and RA. In the synovial membrane of RA, there are abundant neutrophils that locally release NETs, so that RA patients' circulation can show an increase in NETs [76] . In addition, peptidyl arginine deaminase 4 (PAD4), a calcium-dependent enzyme located in the nucleus of neutrophils, accelerates histone citrullination and promotes the formation of ACPA. The autoimmune antibody complexes promote RA inflammation by stimulating neutrophils to form more NETs, activating the formation of FLS and secreting IL-6 and IL-8 [77]. Veras et al. [78] studied 32 hospitalized patients infected by the SARS-CoV-2 virus and found that COVID-19 patients had elevated concentrations of NETs in plasma, tracheal secretions and lung tissue compared to people without COVID-19. Furthermore, a cohort follow-up found that the level of NETs decreased in patients who improved and recovered, providing the possibility for NETs as a potential detection marker for assessing disease progression in patients with COVID-19 [79].

### 3.2 Role of ACE2 receptor in both COVID-19 and RA pathogenesis

Renin-angiotensin system (RAS) is an important humoral regulatory system consisting of peptide hormones and corresponding enzymes [80]. ACE2, a type I transmembrane protein, is capable of regulating the RAS and converting harmful Ang II to beneficial Ang-(1-7), which binds to Mas receptors to antagonize the classical RAS pathway and play a role in vasodilation, maintaining blood pressure, antioxidant stress, anti-inflammatory, and anti-fibrosis [81]. As is known, ACE2 is considered to be an essential functional receptor for the fusion of SARS-CoV-2 with host cells. SARS-CoV-2 binds to ACE2 receptors after its S protein is hydrolyzed and cleaved by recombinant transmembrane protease, serine 2 (TMPRSS2), promoting virus endocytosis, replication, and intercellular transmission [82, 83]. Furthermore, it has been shown that the affinity of ACE2 for the receptor binding domain of SARS-CoV-2 S protein is elevated 10-20-fold compared to SARS-CoV, which may explain the higher incidence of SARS-CoV-2 infection [81, 84]. Previous studies on coronaviruses found that ACE2 protein was downregulated after the entry of the virus, resulting in an imbalance in ACE/ACE2 regulation. Subsequently, this led to elevated levels of Ang II and excessive stimulation of Angiotensin II Type 1 Receptor (AT1R), causing severe pulmonary edema and an increased inflammatory response [85]. Meanwhile, decreased ACE2 levels may also lead to activation of the des-Arg^9^-Bradykinin (DABK) pathway, further exacerbating symptoms and amplifying lung inflammation and injury [86]. ACE2 is beneficial for RA, promoting the production of Ang-(1-7), which can reduce the release of cytokines, the aggregation of white blood cells, vascular density, tissue damage, and fibrosis [80]. Some studies have found increasing levels of Ang II and ACE in plasma, as well as an increasing ACE/ACE2 ratio in RA patients [87]. This suggests that an imbalance of ACE/ACE2 may occur in the pathogenesis of RA, leading to increased Ang II levels and stimulated immune cells secreting more inflammatory factors at the infection sites [88]. Additionally, it promotes the production of local VEGF in angiogenesis, thereby promoting the occurrence of RA [89].

Interestingly, patients with RA and COVID-19 have a higher risk of developing co-morbid cardiovascular disease compared to the general population [80, 90]. As ACE2 receptors are present in cardiomyocytes, many studies have suggested that the heart is a target organ for this virus and may be a significant cause of myocarditis and myocardial injury [91]. Chen et al. [92] identified that pericytes of cardiac vessels expressing ACE2 in large numbers may be the target cells of SARS-CoV-2. If the virus infects the heart, it can affect capillary endothelial cell function and cause microvascular dysfunction. What's worse, it has also been found that myocardial ACE2 expression is significantly increased in heart failure patients, making them more vulnerable to cardiac infection with SARS-CoV-2 and possibly further cardiac injury. More importantly, ACE2 receptors are abundant on the surface of type 2 alveolar epithelial cells. This virus can cause severe impairment of lung function, leading to clinical symptoms such as respiratory dysfunction, hypoxemia, shock, or hypotension. These symptoms often result in insufficient myocardial oxygen supply, further promoting myocardial cell damage [91]. Importantly, Ang II in RAS and other thrombotic components such as DABK and Lys-Des-Arg^9^-BK (DAKD) in the Kallikinase-Kallikin System (KKS) can be degraded by ACE2, but downregulation of ACE2 in COVID-19 can promote thrombosis and increase the risk of myocardial infarction [93]. As known, RAS can regulate electrolyte balance that is often disrupted in both COVID-19 and RA, resulting in electrolyte disorders and hypokalemia [94]. In a study on the relationship between RA and atherosclerosis, it was found that the levels of Ang II, Ang-(1-7), and ACE in RA patients' plasma were increased, while the concentration of ACE-2 was negatively correlated with carotid intima-media thickness, indicating RAS activation in RA patients with associated cardiovascular risk [87].

### 3.3 Relationship between inflammation and cardiovascular complications

Acute cardiovascular inflammation can repair heart damage, but uncontrolled chronic inflammation can lead to heart damage. Studies have shown that inflammation promotes the development of cardiovascular diseases, including myocardial infarction [95], atherosclerosis [96], and heart failure [97]. Inflammatory response is also an important link in COVID-19, RA, and cardiovascular complications. Studies showed high density of CD68+macrophages and CD3+lymphocytes in myocarditis patients infected with SARS-CoV-2, indicating that there may also be infiltration of inflammatory cells in COVID-19 patients’ myocardium [98]. Neutrophil lymphocyte ratio (NLR) is a novel inflammatory marker that has been studied as a predictor of the occurrence and progression of cardiovascular diseases, such as acute heart failure [99, 100], and arrhythmia [101]. Several studies have found that severe COVID-19 patients often have an increased NLR [34, 102], indicating an increased risk of cardiovascular disease and poor prognosis. Studies have found that virus infection can cause a cytokine storm that can lead to complications such as myocardial injury and heart failure through an imbalance of Th1/Th2 cells [91, 103]. COVID-19 and RA produce high levels of proinflammatory cytokines during the disease process, which may lead to vascular endothelial cell damage and are the pathological basis for the process of cardiovascular disease. Elevated levels of inflammatory cytokines, such as TNF-α, IL-6 [104], and IL-17A [105], can induce endothelial dysfunction, as shown in many studies. Endothelial dysfunction leads to increased vascular permeability and promotes the expression of cell surface adhesion molecules, chemokines, as well as prethrombotic mediators, promoting the formation of thrombosis [106] and atherosclerosis [107]. Additionally, overactive NETs in COVID-19 and RA can induce platelet activation and promote thrombin production by expressing functional tissue factors, causing pathogenic immune thrombosis and myocardial infarction, which may be one of the causes of COVID-19 morbidity and mortality [79, 108].

## 4. Challenges in Treating Patients with COVID-19 and RA

### 4.1 Impact of immunosuppressive therapies on COVID-19 outcomes

RA patients commonly use DMARDs to manage their disease [59]. Some DMARDs belong to the class of immunosuppressive drugs, which can lead to immunosuppression within the body. This is of particular concern when treating COVID-19 infection in RA patients due to their immune dysfunction and potential comorbidities [109]. While immunosuppressants can reduce cytokine storms triggered by TNF, IL-1, IL-6, and interferon, which may mitigate serious organ damage in COVID-19 patients [110], they also increase the risk of infections due to a reduced autoimmune response. Studies have shown that immunosuppressant therapy may worsen COVID-19 outcomes, compared to patients receiving cytokine inhibitor therapy [36, 37]. In addition, RA can worsen due to infection through the iatrogenic effects of immunosuppressants [111]. Glucocorticoids (GCs) are commonly used immunosuppressive medications in RA treatment and have been found to alleviate high inflammatory state in advanced COVID-19 cases, reducing mortality and hospitalization time [112, 113]. However, excessive use of GCs can inhibit immunity and delay virus clearance. Patients with rheumatism using GCs (≥10 mg prednisone daily) have a higher probability of hospitalization due to severe COVID-19 [40]. In addition, the use of GCs may accelerate bone loss in COVID-19 patients, especially older patients, who are more susceptible to osteoporosis [114]. A clinical study involving individuals with chronic inflammatory arthritis found that low-dose GCs were associated with an increase in COVID-19 [115]. Research has also suggested that patients with RA taking bDMARDs are more likely to develop COVID-19 than those taking csDMARDs [116] , and this risk is increasing over time [117]. In summary, the use of immunosuppressants in treating COVID-19 in RA patients presents a double-edged sword. Clinicians must weigh the anti-inflammatory benefits against the possible side effects based on each patient's specific situation.

### 4.2 Management of comorbidities in patients with RA and COVID-19

The comorbidities associated with RA and COVID-19 depend on the severity of the patient's disease. RA has comorbidities with symptoms similar to COVID-19, including obesity, diabetes, hypertension, and other chronic diseases that can exacerbate each other [109, 118]. Research has shown that low-grade inflammation caused by obesity (BMI ≥ 30 kg/m^2^) can lead to abnormal secretion of cytokines, adipokines, and interferon in COVID-19 patients, further impairing the immune response [119, 120]. Li et al. analyzed a sample of 353,299 England and found that metabolically unhealthy obesity and vitamin D insufficiency are associated with a significantly increased risk of COVID-19 severity, especially for adults 65 years of age and older, indicating that vitamin D status might moderate the associations between metabolic/ obesity phenotypes and COVID-19 outcomes [121]. Similarly, RA patients, especially those who are very obese, produce more inflammatory proteins and exacerbate arthritis. For patients with obesity, treatment should focus on reducing weight and increasing medication doses [122]. Diabetes is also a possible comorbidity of both diseases and is affected by the chronic inflammatory state. The comorbidity of obesity may also affect diabetes in patients with COVID-19 and RA [123]. Several studies have shown that SARS-CoV-2 can directly damage the pancreas, aggravating hyperglycemia and leading to diabetes attacks in previously non-diabetic individuals [124]. Moreover, the potential prethrombotic state can further exacerbate the overactivation of coagulation cascades in COVID-19 with diabetic patients [125]. Control of blood sugar in diabetic patients treated for COVID-19 may be negatively affected. For example, corticosteroids, azithromycin, and other therapeutic drugs may increase blood sugar levels and lead to blood sugar disorders [126]. Studies have also shown that the activation of inflammatory mediators, such as cytokines, and later stress states, such as "inflammatory storms" led by severe COVID-19, can aggravate hypertension and cardiac load, significantly increasing the difficulty of blood pressure management and the risk of mortality for patients [127]. Early COVID-19 requires antiviral treatments, but antiviral drugs interact with antihypertensive drugs, necessitating a reduction in the dosage of antihypertensive drugs. Hypotension therapy may also lead to possible adverse events in COVID-19 patients, particularly regarding inflammatory stress, microcirculation disorders, and lung function. Early control of blood pressure and prevention of hypertension emergencies and complications based on mechanisms are essential. Importantly, age is an interfering factor that affects the severity of both diseases. Studies have found that the expression of ACE2 and TMPRSS2 in immune inflammatory stromal cells is related to age [128]. The elderly are more susceptible to severe COVID-19 [129]. For geriatric patients, medication should be used cautiously, and personalized treatment is recommended [130], due to the aging of cellular functions in their bodies.

### 4.3 Risk of medication interactions and adverse effects

As commonly used csDMARDs for the treatment of RA, both chloroquine and hydroxychloroquine were initially thought to be able to resist COVID-19 [131, 132]. However, patients who use hydroxychloroquine or chloroquine as an anti-RA drug for a long time have the same incidence of novel coronavirus infection as those who do not use hydroxychloroquine [133, 134]. Furthermore, it may cause cardiac arrest and sudden death, especially for those who receive both azithromycin and oseltamivir treatment. The use of chloroquine may result in an additive and potentially dangerous QT prolongation [135]. Non-steroidal anti-inflammatory drugs (NSAIDs) are commonly used anti-inflammatory drugs to treat RA. Currently, it has been shown that the use of ibuprofen can induce overexpression of ACE2 in diabetic rats [136]. Since fever is a main symptom of COVID-19, taking NSAIDs may mask fever and prevent timely detection and proper treatment. Additionally, methylprednisolone has been associated with electrolyte imbalance, fluid accumulation, and hypertension [137].

## 5. Potential therapeutic approaches for COVID-19 and RA

### 5.1 RA medication for COVID-19 treatment

As mentioned above, the cytokine imbalance in COVID-19 is comparable to that of RA. Some medications used for RA treatment can directly inhibit certain cytokines or signaling pathways like JAK inhibitors, thus reducing the pro-inflammatory network of RA. Therefore, these drugs may become potential targets to treat COVID-19.

It is important to note that IL-6 is a key inflammatory mediator in autoimmune inflammation and cytokine storms [138], and its high expression is correlated with the severity of COVID-19 and mortality [139]. Thus, IL-6 inhibitors may help to reduce the severe inflammation associated with severe COVID-19 (Fig. 2).Tocilizumab, the first pharmacological anti-IL-6 receptor authorized for RA treatment, initiated two days before intensive care unit admission, was linked to a reduced chance of mortality, as shown in a large cohort analysis [140]. Meanwhile, the injection of tolimumab substantially relieves clinical symptoms in COVID-19 patients with severe/critical lung illness [141-143]. Sarilumab, another anti-IL-alpha receptor antibody used to treat RA that has not responded adequately to methotrexate or csDMARDs, was found in a recent study to reduce the risk of 6-month mortality by more than 99.9% when used in the therapy of severely ill COVID-19 patients [144]. More data from ongoing randomized controlled trials are needed to understand the impact of IL-6 inhibitors in the management of COVID-19.

Anakinra (ANK) is a recombinant human IL-1 receptor blocker (Fig. 2) that can reduce cytokine storms by inhibiting IL-1 production [145]. In the first trial of anakinra for COVID-19 patients with RA, high doses of anakinra were found to be safe and associated with better respiratory function by reducing cytokine storm levels in 72% of patients [144]. ANK has also been shown to lower the likelihood of invasive mechanical ventilation and mortality in the ICU [146]. Furthermore, reducing IL-1 levels decreased endothelial cell injury and microangiopathy [147]. Additional studies are needed to further explore the effectiveness and safety of anakinra in COVID-19 patients.

TNF is also a major pro-inflammatory modulator in RA, and it has been linked to pulmonary and vascular tissue injury, coagulopathy, and other symptoms [148, 149]. High levels of TNF in COVID-19 patients' plasma and tissues are associated with poor prognosis and early mortality [150, 151]. TNF inhibitor treatment reduces the amounts of IL-6 and IL-1 and stops leukocytes from moving to areas of inflammation [152]. Currently, TNF inhibitors like infliximab, etanercept, adalimumab, and others are often used to treat RA (Fig. 2). Interestingly, hospitalization rates were lower in COVID-19-infected RA patients who received therapy with TNF inhibitors [119]. A study conducted on COVID-19 patients receiving infliximab has revealed a drop in serum IL-6 as well as a decrease in deaths [153]. More clinical studies are needed to further evaluate their efficacy.

Currently, there are five types of JAK inhibitors clinically used for RA, such as baricitinib, tofacitinib, upadacitinib, peficitinib, and filgotinib [154]. These inhibitors can block the transmission of biological signals by tyrosine kinases, thus blocking the role of various inflammatory factors, and reducing the inflammatory response, making them promising potential therapeutic targets against COVID-19 (Fig. 2) [64]. When SARS-CoV-2 enters host cells via ACE2-mediated endocytosis, it is regulated by proteins like AP2-associated protein kinase 1 (AAK1) [155]. A study found that baricitinib, a drug used to treat moderate and severe RA patients, can effectively prevent SARS-CoV-2 from entering normal cells by combining with AAK1. Additionally, this drug reduces lung inflammatory injury by decreasing intracellular levels of several pro-inflammatory signals such as IL-6, IL-12, IL-23, and IFN-γ, as well as cytokine storms [156]. In an extension analysis of the COV-BARRIER trials, 101 individuals with COVID-19 were compared to the efficacy of baricitinib versus placebo. It was found that baricitinib reduced death rates by 46% at 28 days and 44% at 60 days [157]. A recent study also found that it substantially decreased the risk of death or respiratory failure in patients hospitalized with COVID-19 [158]. However, it has been noted that JAK inhibitors like ruxolitinib, baricitinib, and tofacitinib may increase the likelihood of developing mild viral respiratory symptoms [159]. The chance of herpes zoster recurrence is also increased in people who take JAK inhibitors [160]. Given these side effects, it is necessary to conduct additional studies to further evaluate their involvement in COVID-19.

Theoretically, glucocorticoids play an important role in inhibiting lung inflammation, especially during advanced illness [161]. Previous studies have shown that low doses of glucocorticoids can inhibit the transcription of some common proinflammatory cytokines, stopping the prolongation of cytokine reactions and hastening the resolution of lung and systemic inflammation [162]. A meta-analysis of 1,703 individuals who suffered from severe COVID-19 revealed that taking dexamethasone greatly decreased all-cause mortality in 28 days [163]. While glucocorticoids have certain immunosuppressive effects that may reduce virus clearance and accelerate virus replication [161], a current investigation of individuals with mild to severe COVID-19 revealed no effect of GCs on viral clearance [164]. Several retrospective studies investigated the administration of methylprednisolone in people who have severe COVID-19 and found that receiving methylprednisolone treatment is linked with lower mortality in ARDS patients [112, 113]. As a result, after SARS-CoV-2 exposure, larger doses of glucocorticoids may be required in severe, organ-threatening diseases [165]. Thus, further research is required to confirm the precise roles of glucocorticoids in the long-term prognosis of COVID-19 patients. Despite this, the WHO recently recommended that glucocorticoids may be the best option for people with severe COVID-19 infection, but not for those who have milder infection manifestations [166]. For RA patients, glucocorticoids can be used at a low dose, and abrupt discontinuation of glucocorticoids should be avoided, even if they exhibit indications of COVID-19 infection [165]. NSAIDs may provide therapeutic antipyretic and/or anti-inflammatory benefits in mild cases of COVID-19, but in patients with severe COVID-19 manifestations, including kidney, heart, or gastrointestinal damage, NSAIDs should be discontinued [34, 167, 168]. Given the severe toxic side effects of hydroxychloroquine, the WHO does not recommend its use in treating COVID-19 [166].

### 5.2 New therapies targeting shared mechanisms

Both angiotensin-converting enzyme inhibitors (ACEIs) and angiotensin II receptor antagonists (ARBs) have strong dual effects on the renin-angiotensin-aldosterone system (RAAS). Thus, they can be used not only for circulatory protection but also as anti-inflammatory and immune modulators [169]. There was concern that these two may worsen the condition by upregulating ACE2 [170], but this has not yet been proven. A series of observational studies and meta-analyses concluded that neither ACEIs nor ARBs increased the risk of SARS-CoV-2 infection, exacerbation of illness, or death [171-174]. Besides, it has also been found that the use of RAAS inhibitors might be preventative against complications and death in COVID-19 patients compared to other antihypertensive drugs [175]. Given that COVID-19 patients often have hypertension, and that the complications caused by discontinuing these drugs may be far worse than the speculated adverse reactions, hypertensive patients are recommended to continue taking RAAS inhibitors.

Statins have potent anti-inflammatory and immunomodulatory effects and are frequently prescribed alongside ACEIs and ARBs for patients with hypertension or cardiovascular disease [176]. Statins also protect endothelial cells and decrease thrombosis and pulmonary vascular injury, so they may help with COVID-19 disease [177]. A retrospective analysis of 13,981 COVID-19 patients in 21 hospitals showed that statin use reduced mortality compared to the non-statin group. Additionally, it substantially reduced the chance of invasive mechanical ventilation, ICU admissions, and ARDS in COVID-19 patients, consistent with a recent meta-analysis [178]. Nevertheless, more research is still needed to determine the precise effects of statins on COVID-19.

Remdesivir (RDV) is an adenosine analogue with antiviral activity that is presently being developed for the treatment of Ebola virus infection [179]. A recent experiment showed that rhesus monkeys treated with RDV showed no signs of respiratory illness, and lung infiltration on X-ray films was lower than in animals treated with vectors. The pulmonary viral load of animals treated with RDV was substantially reduced at necropsy on the seventh day after inoculation, as was lung tissue damage [180]. Beigel et al. [181] published the findings of a randomized, double-blind, placebo-controlled clinical study of RDV treatment for COVID-19. In their study, 538 patients were given RDV and 521 were given a placebo, and the former had higher clinical healing rates than the latter. These results revealed the potential therapeutic effect of RDV. Paxlovid is an anti-novel coronavirus drug packaged in a combination of nirmatrelvir/ritonavir. According to the EPIC-HR clinical research, patients treated with Paxlovid within three days of symptom onset had an 89% decreased likelihood of hospitalization and death compared to placebo (https://bit.ly/3sTNGqh). But it can also cause hypertension and damage to the liver and kidneys, as well as other side effects and medication contraindications. It is primarily intended for the treatment of patients aged ≥12 years, weighing ≥40 kg, with mild to moderate COVID-19 who have high-risk factors for progression to severe disease. In these patients, the Infectious Diseases Society of America (IDSA) guidelines recommend starting the drug within 5 days after the onset of symptoms (https://mxnzp.com/sl/AaIC).

### 5.3 Role of vaccination in preventing COVID-19 in patients with RA

There are three types of COVID-19 vaccines available: inactivated, recombinant subunit protein, and viral vector. Clinical trials have shown that these vaccines can stimulate both cellular and humoral immune responses, leading to a reduction in the severity of COVID-19. However, there are some new and comparatively more contagious variants that have aroused people’s concern, because some mutations may cause a resistance to vaccine-elicited serum and antibodies [182]. Whether existing vaccines can effectively respond to mutant strains still needs to be monitored. But vaccination should be for all, for the rapid elimination of the worldwide pandemic situation, despite their age [183]. Studies have indicated that the immunogenicity of the COVID-19 vaccine is reduced after treatment with rituximab (RTX), abatacept, methotrexate (MTX), and glucocorticoids, even in patients with RA. Therefore, it may be beneficial to administer a higher vaccine dose or even a third or fourth dose of the mRNA vaccine, and to monitor the patient's immunity continuously [184]. Some studies suggest that discontinuing anti-RA drugs for two weeks before administering the COVID-19 vaccine can effectively increase antibodies against the SARS-CoV-2 virus in RA patients [185]. Combination therapy with DMARDs and prednisone has no effect if MTX treatment is temporarily stopped for patients. After three doses of the COVID-19 vaccine, RA patients still exhibit a positive response, even if they are receiving low-dose RTX. Thus, the best vaccination strategy may be to administer the vaccine as late as possible after the lowest RTX dose for RTX treatment. Once serum transformation occurs, the positive reaction will persist, even if RTX is still used [186]. This suggests that RA patients receiving RTX may be at an increased risk of severe COVID-19 [187]. The reason for this may be that RTX depletes B cells, leading to a reduction in protective salivary IgG, potentially impairing the body's ability to fight off airborne pathogens [188]. Therefore, other DMARDs may be better options than RTX [189].

## 6. Conclusion

In conclusion, there are similarities in the immune responses and cytokine disorders during the development of COVID-19 and RA. Both diseases involve JAK-STAT signaling pathways and increased pro-inflammatory cytokines such as IL-6, TNF-α, IL-1β, IL-17, and GM-CSF. ACE2 function is also relevant in both COVID-19 and RA. After SARS-CoV-2 enters the host cell via the ACE2 receptor, it can upregulate Ang II levels by regulating RAS, exacerbating the inflammatory damage within lung tissue, which is also linked to the onset of RA. Importantly, the inflammatory reaction underlying COVID-19 and RA may promote the occurrence of thrombosis, myocardial infarction, and atherosclerosis. Therefore, immunosuppressive therapies have the potential to reduce cytokine storms, but they may also increase the risk and severity of SARS-CoV-2 infection in RA patients. Certain drugs, such as chloroquine/ hydroxychloroquine, have shown promise, but their use in treating COVID-19 must be modified due to adverse outcomes and/or side effects caused by drug interactions. Recognizing the role of pathological hyperinflammatory states in driving the development of COVID-19 and RA pathology, certain biological agents for RA treatment, such as IL-6 inhibitors (tocilizumab, sarilumab), IL-1 inhibitors (anakinra), TNF inhibitors (infliximab, adalimumab), and tsDMARDs like tofacitinib and baricitinib, may help improve COVID-19 outcomes. New antiviral medications, such as Remdesivir, are also expected to be further studied and used in COVID-19 treatment.

Although studies on anti-rheumatic drugs have shown their therapeutic efficacy in COVID-19 patients, more clinical trials are needed to determine their precise therapeutic action in RA patients with COVID-19. Considering that immunosuppressive therapies may reduce COVID-19 vaccine antibody responses and that variant virus strains alter infection dynamics and severity, immunosuppressive therapy should be closely monitored. More studies are needed to determine how it can affect COVID-19 prognosis and the development of new therapies targeting the immune-inflammatory mechanisms shared by RA and COVID-19.
